# PKC in Regenerative Therapy: New Insights for Old Targets

**DOI:** 10.3390/ph10020046

**Published:** 2017-05-18

**Authors:** Marta Rui, Rita Nasti, Emanuele Bignardi, Serena Della Volpe, Giacomo Rossino, Daniela Rossi, Simona Collina

**Affiliations:** 1 Department of Drug Sciences, Medicinal Chemistry and Pharmaceutical Technology Section, University of Pavia, Viale Taramelli 12, 27100 Pavia, Italy; marta.rui01@universitadipavia.it (M.R.); rita.nasti01@universitadipavia.it (R.N.); emanuele.bignardi01@universitadipavia.it (E.B.); serena.dellavolpe01@universitadipavia.it (S.D.V.); giacomo.rossino01@universitadipavia.it (G.R.); daniela.rossi@unipv.it (D.R.); 2 Centre for Health Technologies (CHT), Department of Drug Sciences, Medicinal Chemistry and Pharmaceutical Technology Section, University of Pavia, Viale Taramelli 12, 27100 Pavia, Italy

**Keywords:** regenerative medicine, chronic or non-healing wounds, protein kinase C (PKC), re-epithelization, PKC ligands

## Abstract

Effective therapies for chronic or non-healing wounds are still lacking. These tissue insults often result in severe clinical complications (i.e., infections and/or amputation) and sometimes lead to patient death. Accordingly, several research groups have focused their efforts in finding innovative and powerful therapeutic strategies to overcome these issues. On the basis of these considerations, the comprehension of the molecular cascades behind these pathological conditions could allow the identification of molecules against chronic wounds. In this context, the regulation of the Protein Kinase C (PKC) cascade has gained relevance in the prevention and/or reparation of tissue damages. This class of phosphorylating enzymes has already been considered for different physiological and pathological pathways and modulation of such enzymes may be useful in reparative processes. Herein, the recent developments in this field will be disclosed, highlighting the pivotal role of PKC α and δ in regenerative medicine. Moreover, an overview of well-established PKC ligands, acting via the modulation of these isoenzymes, will be deeply investigated. This study is aimed at re-evaluating widely known PKC modulators, currently utilized for treating other diseases, as fruitful molecules in wound-healing.

## 1. Introduction

Regenerative medicine is a multi-approach branch of translational research, involving both reparative and regenerative strategies, with the aim to restore the normal functions of damaged tissues. Wound healing in particular represents an important target of regenerative medicine [1]. Accidental traumas and/or surgery are the main causes of wounds, even if chronic wounds are often related to other pathological conditions, i.e., cancer or diabetes [2]. When an injury occurs, the human body promotes a dynamic process consisting of consecutive phases of inflammation, cell proliferation and maturation, thus providing wound repair [3]. From a microscopic standpoint, damage triggers a series of molecular cascades that collimate into self-repair processes; nonetheless, lesions such as chronic or non-healing wounds (e.g., vascular insufficiency ulcers, diabetic ulcers, pressure sores and radiation necrosis) do not activate these natural reparative mechanisms [4]. Accordingly, such conditions often result in severe clinical complications (e.g., infections and/or amputation) and sometimes lead to patient death; therefore, regenerative medicine therapies may represent powerful strategies to circumvent these issues [5]. Nevertheless, the significant percentage of morbidity and relapses, as well as the high risk of treatment failure, render urgent the need to identify advanced therapies, aimed at improving the outcome of related conditions and the quality of life of affected patients [6].

The past few years have seen a growing interest in drugs and technologies with the potential to regenerate and to repair tissues [7]. Related research approaches are numerous, ranging from treatments with single molecules or peptides to the engineering of entire organs and so far novel and, at the same time, highly informative mammalian models for wound healing have been developed [8,9]. Several biochemical pathways have been studied for identifying potential targets against chronic wounds and new small molecules are currently under investigation [9].

**Figure 1:**
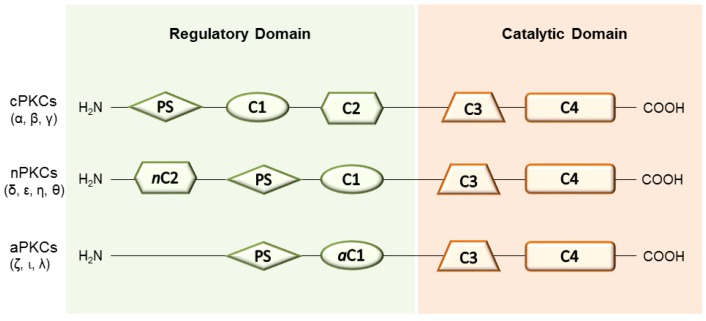
General structure of PKCs.

In the context of prevention and/or reparation of tissue damages, the activation of the diacylglycerol (DAG)-protein kinase C (PKC) cascade has gained certain relevance [10] and the role of PKCs in several physiological and pathological processes has been widely documented [11,12]. The PKC protein family is constituted of serine/threonine phosphorylating enzymes whose activation via second messenger has direct involvement in the regulation of numerous cellular functions (i.e., differentiation, metabolism and apoptosis) [13,14]. Ten well-characterized full-length mammalian isoenzymes have been discovered and grouped into three classes, based on their structural features and sensitivity to activators: (i) conventional or calcium-dependent cPKCs (α, βI, βII and γ); (ii) novel or calcium-independent nPKCs (δ, ε, η and θ); (iii) atypical aPKCs (ζ, ι and λ) [15,16] (Figure 1). Structurally, the polypeptide chain of PKCs presents four conserved domains (C1–C4) linked by a hinge region. In detail, the N-terminal regulatory region includes the C1 and C2 domains, which control the kinase activity of the enzyme as well as its subcellular localization. On the other hand, the C3 and C4 domains form the C-terminal catalytic region and they are broadly known to bind adenosine-5’-triphosphate (ATP) and substrate proteins, respectively [17,18,19]. Both endogenous and exogenous activators of PKCs have been identified so far. In particular, diacyl glycerol (DAG) and related phorbol esters display high affinity towards the C1 domain (respectively, related subdomains C1a and C1b) of cPKCs and nPKCs, whereas anionic lipids bind the C2 domain in a Ca^2+^-dependent manner (only cPKCs). Still, atypical isoenzymes (aPKCs) are unable to bind either DAG or Ca^2+^ and they possess a peculiar mechanism of activation involving the formation of protein-protein structures [20]. Besides, all PKCs present a pseudo-substrate sequence (PS), which maintains the protein in an inactive state, further regulating enzyme activation [17,21,22].

**Figure 2:**
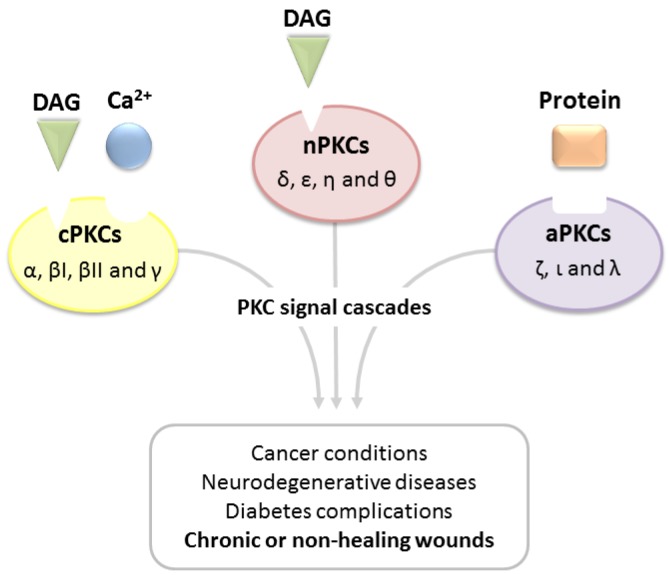
Alterations of PKC signal cascades promote several diseases.

Upon binding of activators, PKCs translocate to the plasma membrane, providing an interaction with the phospholipidic bilayer. This event results in the pseudo-substrate release from the catalytic site, thus activating the enzyme [21,22]. Considering the broad involvement of PKCs in fundamental cell mechanisms, alterations in their signaling cascade may contribute to the etiology of several diseases (Figure 2) [23,24,25,26,27,28]. Accordingly, an overexpression of cPKC isoforms in breast, liver, kidney and prostate cancers has been well documented, whereas high levels of aPKCs have been identified as hallmark of malignant lung carcinoma [29,30,31,32,33]. It is worth noting that recent studies have shown that PKC isoforms involved in tumor genesis are actually inactivated mutations (mainly *loss-of-function* (LOF) mutations), thus revealing the importance of PKCs as tumor suppressors [34,35]. Another aspect to take into account is the strict relation between PKCs and neurodegenerative diseases [36,37,38]. In detail, under physiological conditions, these isoenzymes modulate the generation of amyloid precursor protein (APP), promoting the α-secretase activity therefore decreasing the production of amyloid-β proteins, which are the main macromolecular structures involved in Alzheimer’s disease. Nonetheless, a strict balance in the activity of PKCs is needed: in fact, recent findings have suggested the enhanced activity in gain-of-action mutations of PKCα to promote the insurgence of Alzheimer’s disease by reducing synaptic activity through amyloid-β accumulation. [39]. Additionally, recent in vitro and in vivo studies have demonstrated the involvement of PKCs, in particular isoform β, as promoters of diabetic retinopathy, and thus inhibition of this isoform may contribute to erasing this pathology [40].

In this review, we will focus on the emerging role of the PKC protein family in tissue regeneration, which has attracted great attention in the last ten years, and we will draw an overview on historical PKC ligands, from early discoveries to the present. Our aim is to disclose the possible applications of well-established PKC modulators in regenerative therapy.

## 2. PKC Isoenzymes and Their Role in Tissue Regeneration

**Figure 3:**
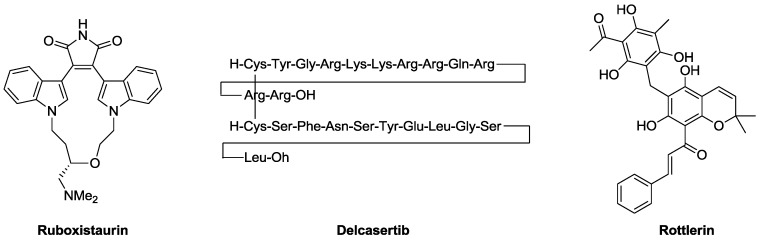
Ruboxistaurin and delcasertib have entered in clinical trials. Rottlerin, a PKCδ (nPKC) selective inhibitor.

As stated in the previous paragraph, PKC isoenzymes are involved in a variety of both physio- and pathological processes and are thus attractive as drug targets. Some compounds, such as ruboxistaurin and delcasertib (Figure 3), targeting different PKC isoforms, have indeed entered clinical trials for diverse pathologies (namely, diabetes and related complications, heart diseases and cancer) [41,42,43]; although promising, most of these molecules failed to complete the clinical development process due to both unfavorable clinical outcomes and unexpected side effects [28].

Concerning regenerative therapy in particular, despite some pioneer studies on the involvement of PKC signaling in regenerative mechanisms carried out in the early 90s, the role of PKC isoenzymes in tissue repair has only started being investigated in depth during the past few years [44]. Recent literature shows that PKC isoforms α (cPKC) and δ (nPKC) are those mainly involved in the regenerative process, especially associated to aforementioned non-healing or chronic wounds secondary to conditions such as neuropathy, peripheral vascular disease and insulin resistance typical of diabetes [45,46]. Herein, we briefly discuss the role of such isoenzymes in re-epitelization and lesion repair, respectively related to two different intracellular mechanisms.

**Figure 4:**
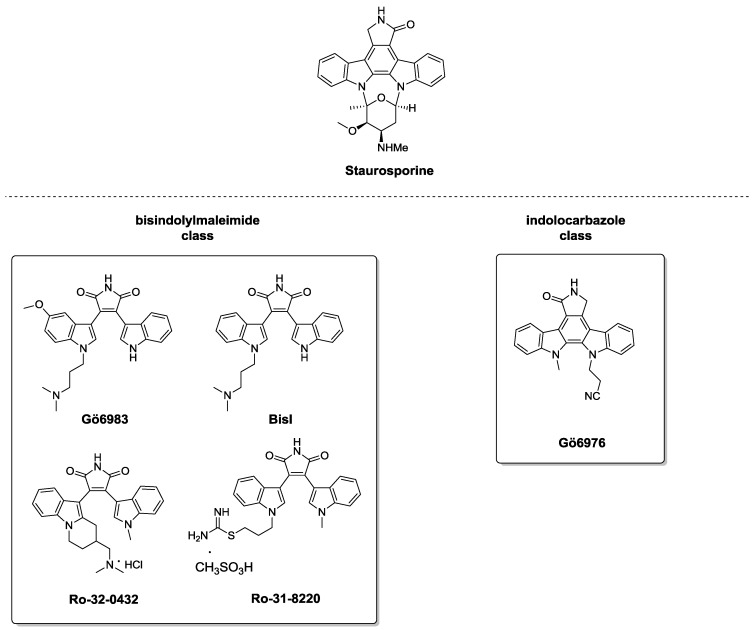
Staurosporine derivatives.

In 2012, the role of PKCα (belonging to the cPKCs) in the regulation of wound re-epithelialization, and particularly their influence in the crucial process of cell-cell adhesion, was demonstrated [45]. In normal epidermis, tissue integrity is assured by cell adhesion complexes (desmosomes), which undergo modifications to guarantee proper repair when wounds occur. In particular, after tissue damage, desmosomes switch from “hyper-adhesive” and Ca^2+^-independent to a Ca^2+^-dependent behavior. This process seems to be regulated by PKCα which, upon translocation to the desmosomal plaque and activation, mediates the conversion to Ca^2+^-dependent desmosomes. The correlation between PKCα and re-epithelization mechanisms was demonstrated through in vitro experiments, where a selective PKCα inhibitor (Gö6976, Figure 4), caused delay in wound closure. In support of this evidence, in vivo study on knockout PKCα mice (PKCα^−/−^) showed that, after incisional lesions, these animals are unable to promote the re-epithelization processes. Moreover, in bistransgenic mice, where the constitutively active PKCα is over-expressed, wound healing presented a two-fold increase compared to wild-type mice. Altogether these results supported the idea that PKCα modulation is a possible strategy for promoting epidermal regeneration [45].

Four years later, the effect of an nPKC, specifically of PKCδ, on wound healing was evaluated [46]. The study performed by Khamaisi et al. focused on the different attitude towards lesion repair of diabetic and healthy fibroblast. The preliminary observation that fibroblasts involvement in tissue regeneration is generally due to their paracrine secretion of crucial molecular mediators, such as angiogenic factors, cytokines and immunomodulators, was the starting point for this investigation [46]. Moreover, the potential of these cells as therapeutic tool, had already brought to fibroblast transplant being proposed as part of regenerative therapy in wounded patients with interesting results [47,48].

This approach was less effective in diabetic subjects, probably owing to the multiple alterations determined by diabetes itself, such as abnormal blood glucose levels, impaired Vascular Endothelial Growth Factor (VEGF) expression levels and PKC activation [49]. Comparing diabetic and healthy fibroblast, Khamaisi studied the involvement of PKC isoforms in wound healing and hypothesized that an altered expression/activation of PKCδ may be responsible for the impaired ability of diabetic fibroblasts in effectively stimulating wound healing as confirmed by in vitro/in vivo analysis [46]. Moreover, treatment with the PKCδ selective inhibitor rottlerin (Figure 3) and with the PKCβ selective inhibitor ruboxistaurin (Figure 3), confirmed that the isoform δ is the mainly involved in wound healing processes, in fact ruboxistaurin failed to reproduce the effects exerted by rottlerin [46]. Altogether, these data suggested the central role of PKCδ in the impairment of healing ability of diabetic fibroblasts. The hypothesis was confirmed in vivo, through transplant in nude mice of either control or diabetic fibroblasts both presenting inhibited PKCδ; such inhibition significantly improved the healing ability upon wounding, as well as increased VEGF expression and neovascularization. Given the final evidence collected through experiments on murine models of diabetes (STZ-induced diabetic mice), the authors concluded by proposing transplant of fibroblasts where activation of PKCδ has been blocked ex vivo as possible therapeutic tool for promoting wound healing in diabetic subjects [46].

To sum up, PKCα and PKCδ seem to be valuable targets for promoting tissue regeneration and ligands selective towards PKCα and PKCδ may then represent innovative drugs for the treatment of chronic or non-healing wounds. To stimulate the interest of medicinal chemists in developing novel selective PKC ligands, in the next section an overview of the most relevant compounds discovered so far will be discussed.

## 3. PKC Ligands

The most common target of PKC ligands is the highly conserved ATP-binding C3 domain, common to several protein kinases and accordingly, molecules active on this site are characterized by lack of selectivity [47]. Differently, PKC regulatory domain C1 constitutes an intriguing pharmacological target for the development of new selective ligands. This domain is tightly related to PKC activation and it is only shared with six other non-PKC small kinase families (PKD, chimaerins, the guanyl nucleotide-releasing proteins (RasGRPs), the Unc-13 scaffolding proteins, the myotonic dystrophy kinase-related Cdc42-binding kinases (MRCKs), DAG kinase (DGK) isoforms β and γ)). In the last decade, numerous efforts for the design of new ligands have focused on domain C1 that displays higher variability among PKC isoforms [50]. Moreover, the co-crystal structure of PKC δ domain C1 with phorbol-13-*O*-acetate (PDB code: 1PTR) has been solved, allowing for a rational drug design approach to access new PKC modulators [50]. Hereinafter, a concise and detailed overview of the principal classes of PKCs α and δ ligands targeting domains C3 and C1 will be reported in chronological order.

### 3.1. C3 Domain Ligands

As previously stated, the majority of current PKC ligands target the catalytic ATP-binding C3 domain; however, since this domain is highly conserved among different protein kinase families, selectivity is still a crucial issue [47,51]. Accordingly, the scientific community has focused its attention on identifying molecules able to selectively interact with the ATP-binding site of PKCs. Among the plethora of well-established PKC C3 domain ligands, Staurosporine (Figure 4) and its derivatives are the most studied [51,52,53]. This natural compound isolated from the bacterium Streptomyces staurosporeus [54] is now commercially available as a potent, non-selective PKC inhibitor. Given its high structural complexity, several research groups have oriented their efforts to designing and synthesizing novel derivatives in the attempt to improve PKC subtype selectivity. In virtue of their chemical structure, they can be grouped into two main classes: (i) bisindolylmaleimides and (ii) indolocarbazoles. Hereinafter, we report the most representative compounds for each group (Figure 4).

Structurally related compounds Gö6983 and BisI, belonging to bisindolylmaleimides, have shown good selectivity for PKC over other kinases, especially towards conventional and novel isozymes (Figure 4) [52,55,56]. Interestingly, they do not interact with closely related PKA and PKD. From a pharmacological standpoint, these PKC inhibitors, endowed with high affinity towards different isoenzymes (pan-PKC ligands), have a crucial role in myocardial dysfunctions, promoting cardio-protective effects. Compounds Ro-31-8220 and Ro-32-0432, designed by Roche, are commercially available as PKC inhibitors and associated literature collected during the past years shows their usefulness as a pro-apoptotic and anti-inflammatory agent respectively [53,57].

Concerning the indolocarbazole class, compound Gö6976 has emerged as a potent and selective inhibitor of conventional PKC isozymes [52,55,58,59]. It possesses a wide spectrum of therapeutic applications, exerting both cytotoxic effects towards cancer cells and and anti-viral action. Moreover, as mentioned in the previous paragraph, Gö6976 activity has been exploited in a study aimed at correlating PKCα and re-epithelialization, demonstrating that inhibition of this peculiar isoform prevents human keratinocyte migration and thus delays tissue repair [45]. The ability of Gö6976 in thwarting cell migration was reported in another study focused on verifying the relation between PKCα and wound-healing. In detail, upon treatment with carbon monoxide, this molecule inhibits murine gastric cell repair [60].

**Figure 5:**
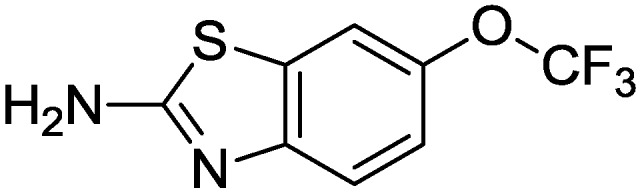
Structure of riluzole.

Another important ligand of the PKC catalytic domain is riluzole (Figure 5), commonly used in the treatment of Amyotrophic Lateral Sclerosis (ALS) [61,62]. In general, the neuroprotective mechanisms associated to riluzole may be ascribed to its antagonistic effect against glutamate receptors [61,62]. Nevertheless, recent studies have highlighted that such compound may inhibit PKC within the catalytic domain, leading to the enancement of the glial glutamate transporter (the excitatory amino acid transporter type 2 (EAAT2)) thus producing antioxidative neuroprotective effect [63].

**Figure 6:**
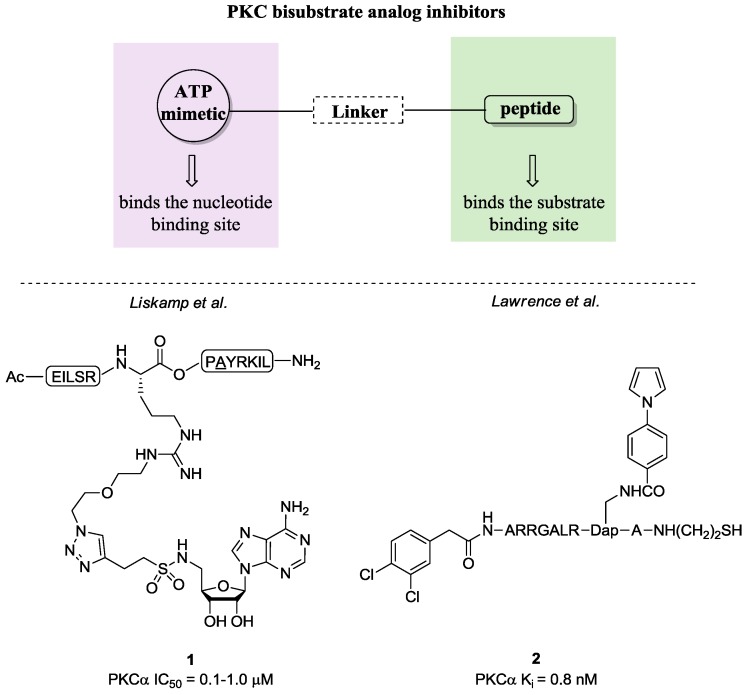
Bisubstrate analog inhibitors.

In the last decade, new selective modulators of the ATP-binding site have been discovered through a mechanism-based approach. The most characteristic class of new ATP-binding site ligands is represented by bisubstrate analog inhibitors. The rationale behind these compounds takes into account the possibility to inhibit PKCs by targeting both C3 and C4 domains [64,65,66,67]. In fact, these molecules present one portion mimicking the phosphate donor region (ATP) and one the acceptor (Ser-, Thr-, or Tyr-containing peptides) bridged by a spacer (Figure 6). Accordingly, these ligands are able to disrupt signal transduction pathways by exploiting two binding sites and even more they may enhance selectivity. Generally, this class of compounds includes sulfonamides, sulfonylbenzoyl, carboxylic acid, dipeptidyl and *N*-acylated peptide, phosphodiester derivatives. In Figure 6, we illustrate two examples highly selective for PKCα (**1** and **2**), which present the described structural features [64,65,66,67].

### 3.2. C1 Domain Ligands

**Figure 7:**
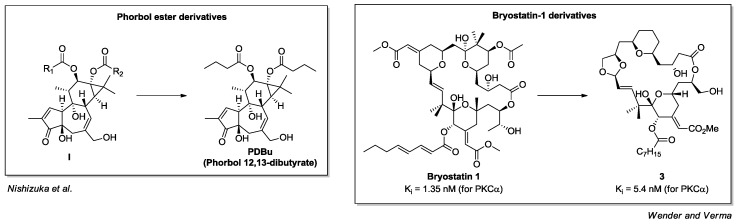
Phorbol ester and bryostatin-1 derivatives.

The most studied non-endogenous-PKC activators targeting domain C1 are the natural compounds phorbol (general structure **I**) and bryostatin-1 [68,69] (Figure 7). Phorbol is a tetracyclic diterpene derived from the plant *Croton tiglium* L. In particular, phorbol 12,13-dibutyrate (PDBu) is an ester derivative with optimized potency and solubility which was employed to prove the importance of PKC in cell proliferation and cancer [70]. On the other hand, bryostatin-1 is a macrolide isolated from marine bryozoan *Bugula neritina* in 1967; considering the low efficiency of bryostatin extraction from its non-renewable natural sources and its challenging synthesis, researchers have produced a series of synthetic simplified analogues [71,72,73,74,75] (Figure 7). Among them, compound **3** has shown interesting selectivity toward novel PKC isoforms (δ, ε) [74].

**Figure 8:**
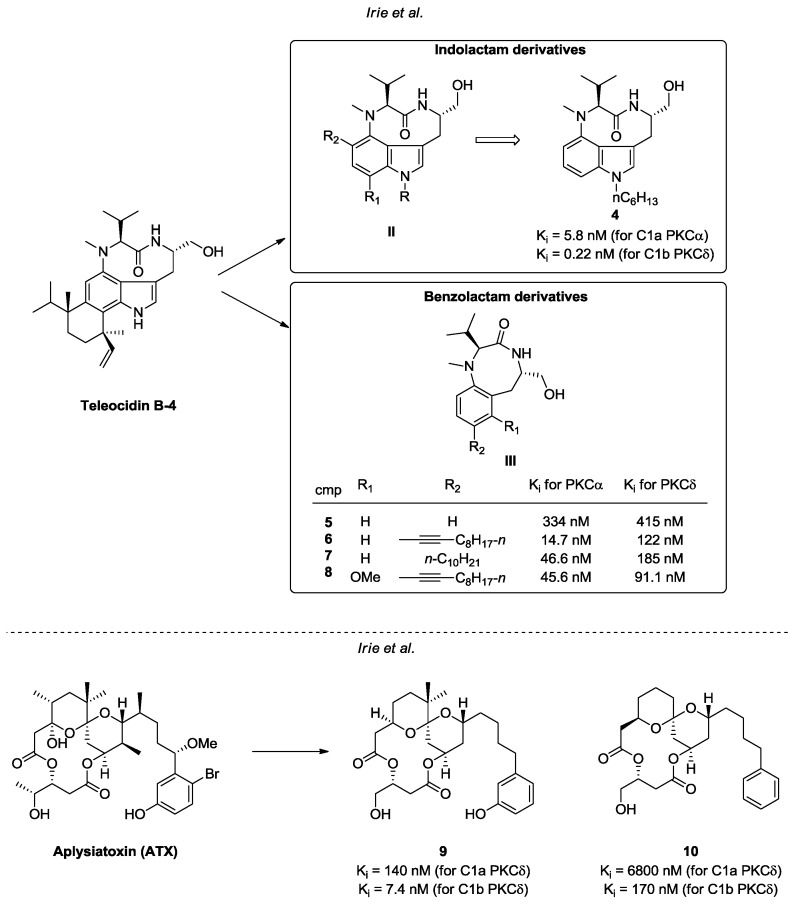
Teleocidin B-4 and ATX derivatives.

Other C1 ligands isolated from natural sources and structurally related to phorbol are teleocidine B-4 and aplysiatoxin (ATX) [76,77] (Figure 8). Regardless of their promising ability to interact with PKC isoforms, the carcinogenic properties of these molecules have harshly limited their utility [78,79]. Several synthetic efforts have been employed to overcome this significant drawback through the development of new derivatives based on these natural scaffolds. In this context, Irie et al. designed and synthetized various indolactam and benzolactam analogues of teleodicin B-4 (general structures **II** and **III**, Figure 8 [80,81,82,83]. Related SAR studies allowed the identification of promising compounds endowed with good affinity and selectivity towards conventional (PKC α, β) and novel isoforms (i.e., PKC δ, ε). Moreover, some of these investigations successfully assessed the compounds affinities towards C1a and C1b sub-domains [80,81,82,83]. The most effective synthetic derivatives of each class (compounds **4–8**) are reported in Figure 8. Concerning ATX derivatives, compounds **9** and **10** (Figure 8) are noteworthy for their antiproliferative properties and their high affinity towards the C1 domain of nPKCs, particularly PKCδ [84].

**Figure 9:**
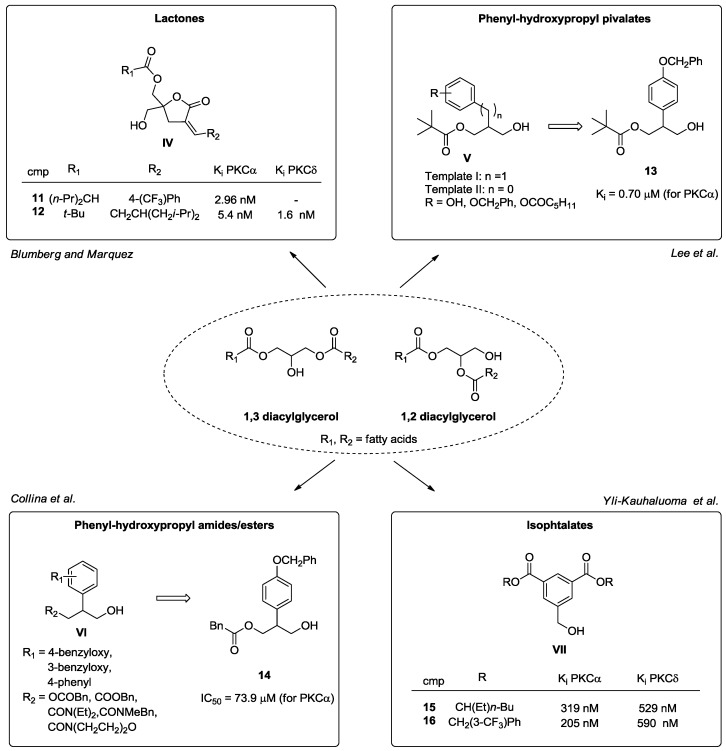
DAG derivatives.

Keeping in mind that DAG is the endogenous substrate of cPKCs and nPKCs, another strategy to develop new C1-ligands exploits the use of DAG derivatives [50]. Accordingly, the group of Blumberg and Marquez synthesized a new series of DAG-lactones (general structure **IV**, Figure 9) with the aim to reduce the entropic penalty associated with the flexible glycerol backbone of DAG [85,86]. Interestingly, the rigid DAG-analogues displayed affinity in the nanomolar range for PKC α and δ, acting as antiproliferative and pro-apoptotic molecules, in the best cases with K_i_ values lower than 10 nM (compounds **11** and **12**). Despite these valuable results, the lack of selectivity over other kinases is still an important issue to consider; in fact, these molecules also showed extremely good affinity towards RasGRPs 1 and 3, due to the presence of the C1 domain [87,88].

In 2006, Lee et al. designed and synthesized 2-phenyl-3-hydroxy propyl pivalates (general structure **VI**, Figure 9), which combined the main pharmacophore features of both DAG and phorbol esters, to identify novel small molecules with high affinity towards PKC α. Related in vitro and in silico assays showed that through the whole series, compound **13** is the most active with a K_i_ value in the submicromolar range [89]. On the basis of these interesting data, our research group synthesized a small library of **13** analogues (general structure **VII**, Figure 9) - ester and amide derivatives—in order to understand which structural modifications on the pivalate template could cause retention or enhancement of affinity towards the C1 domain of PKC. From in vitro and in silico evaluations, compound **14** emerged for its good binding affinities towards PKC α and δ, which resulted comparable to compound **13** [90].

Another class of new synthetic compounds includes isophthalate derivatives (general structure **VII**, Figure 9) designed by Yli-Kauhaluoma et al. through a structure-based approach, easily prepared through four synthetic steps [91]. These molecules are DAG phenylogs, where phorbol ester pharmacophore features are maintained. Biological investigations have disclosed their ability to promote neurite outgrowth via the activation of PKC α with K_i_ values ranging from 210 to 920 nM [91]. In particular, compounds **15** and **16** emerged as the most effective compounds (Figure 9) [92].

## 4. Conclusions

Drugs which benefit wound healing are of high interest to both academia and the pharmaceutical industry. The discovery of effective drugs for the treatment of chronic or non-healing lesions is still a challenge and, as illustrated throughout the present work, further studies on the mechanisms involved in wound healing are still required. Considering the link between PKCα and PKCδ and tissue regeneration pathways, here we propose the identification of novel ligands selective towards PKCα and PKCδ, as a promising strategy for promoting wound healing. The re-evaluation of some well-established PKC ligands already studied for the treatment of different pathologies could be useful as well, as the case-study of riluzole demonstrated. Indeed, riluzole is a well-established drug for treating amyotrophic lateral sclerosis which has been recently proposed for the treatment of diabetic rethinopaty, thanks to its inhibitory effect on PKC.

To fully exploit the potential impact of such approach in wound healing therapy, ad-hoc small molecules should be developed. We believe that the combination of selective PKC modulators with appropriate wound dressing materials could lead to effective therapies able to satisfy the still unmet needs of this area.
